# Finite Element Modeling in Left Ventricular Cardiac Biomechanics: From Computational Tool to Clinical Practice

**DOI:** 10.3390/bioengineering12090913

**Published:** 2025-08-25

**Authors:** Patrick Hoang, Julius Guccione

**Affiliations:** 1 School of Medicine, University of California San Francisco, San Francisco, CA 94143, USA; patrick.hoang@ucsf.edu; 2 Medical Scientist Training Program, University of California San Francisco, San Francisco, CA 94143, USA; 3 Department of Surgery, University of California San Francisco, San Francisco, CA 94143, USA

**Keywords:** finite element modeling, cardiac biomechanics, myocardial infarction, heart failure, ventricular remodeling, hydrogel injections, LVAD optimization, surgical ventricular restoration (SVR), patient-specific modeling, artificial intelligence, machine learning, surrogate models, clinical decision-making

## Abstract

Finite element (FE) modeling has emerged as a powerful computational approach in cardiovascular biomechanics, enabling detailed simulations of myocardial stress, strain, and hemodynamics, which are challenging to measure with conventional imaging techniques. This narrative review explores the progression of cardiac FE modeling from research-focused applications to its increasing integration into clinical practice. Specific attention is given to the mechanical effects of myocardial infarction, the limitations of conventional LV volume-reduction surgeries, and novel therapeutic approaches like passive myocardial reinforcement via hydrogel injections. Furthermore, the review highlights the critical role of patient-specific FE simulations in optimizing LV assist device parameters and guiding targeted device placements. Cutting-edge developments in artificial intelligence-enhanced FE modeling, including surrogate models and precomputed simulation databases, are examined for their potential to facilitate real-time, personalized therapeutic decision-making. Collectively, these advancements position FE modeling as an essential tool in precision medicine for structural heart disease.

## 1. Introduction

The field of cardiac biomechanics encompasses a wide range of topics beyond myocardial mechanics, including valvular biomechanics (mitral, aortic, tricuspid, and pulmonary valves) [1], pericardial [2] and vascular interactions, electromechanical coupling [3], hemodynamics and blood–tissue interactions [4], as well as congenital [5] and acquired structural abnormalities extending beyond the left ventricle (LV). While acknowledging this broader context, this narrative review specifically focuses on FE modeling of LV mechanics and ventricular remodeling, the adaptive structural and functional changes in heart geometry and mechanics following myocardial injury or in response to chronic stress [6].

Finite element (FE) modeling is a computational technique that divides complex biological structures into discrete, smaller elements to predict their mechanical behavior under various physiological conditions [7]. Initially fundamental in orthopedic biomechanics, where FE modeling aids in designing artificial joints, assessing implant stability, and analyzing bone mechanics, benefiting from bone’s relatively rigid structure and minimal deformation before fracture [8]. In comparison, modeling soft tissues, especially cardiac muscle, presents significantly greater complexity. Cardiac muscle experiences considerable deformation, with ventricular myofibers elongating and shortening by approximately 20% during each cardiac cycle [9]. The intricate nature of myocardial tissue, coupled with challenges in determining patient-specific mechanical properties, has considerably impeded the clinical translation of cardiac FE models.

Despite inherent complexities, FE modeling has proven invaluable in elucidating cardiac mechanics, progressively becoming integral to clinical decision-making. While essential traditional experimental studies and imaging methods alone cannot comprehensively represent the heart’s detailed mechanical environment. FE simulations uniquely capture intricate cardiac stress, strain, and hemodynamic patterns, enabling exploration of complex physiological processes, including myocardial deformation, ventricular remodeling, and the mechanical implications of surgical or device interventions. Historically limited to research applications, cardiac FE models are increasingly being incorporated into clinical workflows, facilitating patient-specific treatment planning and optimization [10]. For example, patient-specific FE modeling is increasingly applied to optimize the placement and operating parameters of LV assist devices (LVADs), directly influencing clinical outcomes such as prevention of right ventricular failure [11].

An essential application of FE modeling in clinical cardiology involves evaluating ventricular myofiber stress, crucial for cardiac function and disease progression assessment. Clinicians aim to quantify LV stress distributions to identify deviations from normal values and predict therapeutic outcomes effectively. Aberrant myofiber stress significantly contributes to pathological cardiac remodeling, heart failure (HF) progression, and adverse post-intervention outcomes [11]. Therefore, preoperative quantification of stress distributions is critical for tailoring individualized treatment strategies—be they surgical, pharmacological, or device-based—to maximize both acute and long-term patient outcomes.

The growing integration of FE modeling into cardiac care epitomizes a broader movement toward precision medicine, leveraging patient-specific simulations to refine therapeutic interventions before their execution. Incorporating anatomical and mechanical patient data, FE models provide invaluable insights into the effects of various treatments, such as left ventricular assist device (LVAD) deployment, surgical ventricular reconstruction, or injectable myocardial hydrogels [12]. This predictive capacity facilitates a personalized rather than a generalized treatment approach, significantly enhancing surgical planning and therapeutic outcomes.

However, widespread clinical implementation of FE models faces notable challenges, primarily computational efficiency. Traditional FE simulations demand substantial computational resources, often requiring extensive processing times incompatible with the urgency of clinical decision-making, particularly for critically ill patients [13]. For FE modeling to attain routine clinical utilization, simulations must deliver actionable insights rapidly, ideally within an hour of patient presentation.

To address these computational limitations, researchers increasingly integrate machine learning (ML) and artificial intelligence (AI) methodologies into FE modeling workflows. AI-driven surrogate models, trained using extensive precomputed FE datasets, effectively learn relationships between input parameters—such as myocardial characteristics, loading conditions, and boundary conditions—and resulting model outputs. This approach significantly accelerates the simulation process, reducing analysis durations from hours to mere seconds [13].

Another promising development involves precomputed FE model databases, enabling clinicians to swiftly retrieve simulation outcomes corresponding closely to individual patient anatomies and pathologies. Such databases obviate the need for real-time simulations while preserving the predictive accuracy integral to FE modeling [13].

With ongoing advancements, particularly the integration of AI and database-driven strategies, FE modeling increasingly approaches widespread clinical adoption. This review will elaborate on the clinical translation of these computational models, highlighting their roles in cardiac surgical planning, device optimization, and innovative therapeutic approaches.

**Figure 1:**
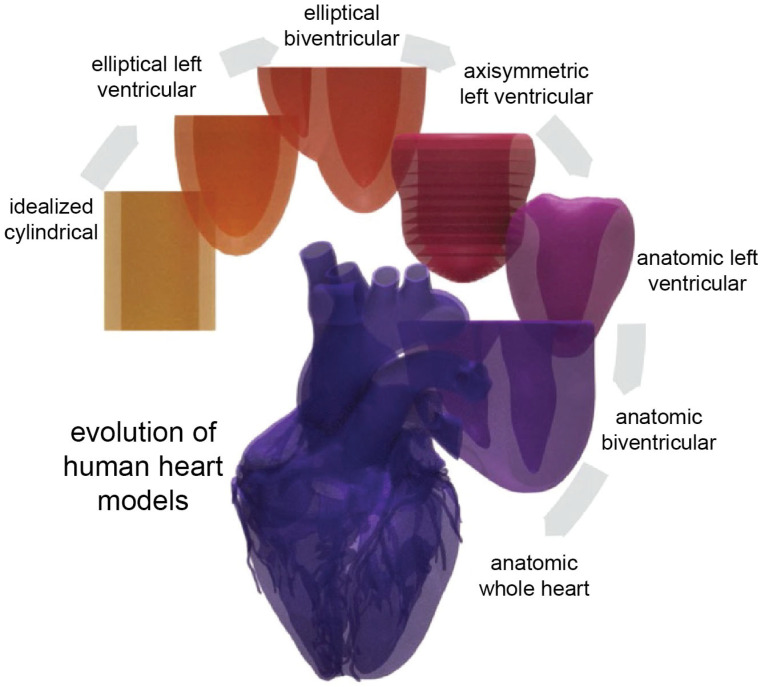
Human heart models have increased in complexity over time. Simplified geometries—such as idealized cylindrical, elliptical LV, and elliptical biventricular models—enable analytical closed-form expressions for fiber orientation. In contrast, more anatomically realistic models, including axisymmetric, LV, biventricular, and whole-heart geometries, are derived from actual human heart anatomy and fiber architecture. Reproduced from [12], under the Creative Commons CC BY 4.0 license.

Supporting this discussion, Figure 1 illustrates the evolution of anatomical cardiac models, demonstrating the progression from simple geometric approximations to detailed, anatomically accurate, anisotropic representations [12]. Such advancements have significantly enhanced patient-specific simulations, directly informing clinical decisions. As FE modeling evolves further, its contribution to cardiovascular medicine is poised to expand, effectively bridging computational research and clinical practice.

The following sections systematically review myocardial infarction’s structural and functional impacts, establish the mechanical basis of HF, and discuss personalized therapeutic strategies guided by FE modeling. Special focus is placed on hydrogel injections as an innovative midwall reinforcement strategy for the LV free wall, emphasizing how FE simulations inform their application and effectiveness. Looking forward, we explore AI-enhanced FE models promising real-time clinical utility through surrogate modeling and precomputed databases. Finally, the review concludes by summarizing essential insights for clinical translation, emphasizing FE modeling’s transformative impact on cardiovascular therapy planning and outlining necessary steps for its broader clinical adoption.

## 2. Structural and Functional Consequences of Myocardial Infarction (MI)

Myocardial infarction significantly contributes to HF, accounting for roughly one-third of all HF cases [14]. While advancements in medical treatments have improved survival rates post-MI, many therapeutic approaches still fail to adequately address the mechanical dysfunction that develops in infarcted and adjacent myocardial tissues. A critical yet frequently overlooked aspect of post-MI remodeling involves diminished contractility within the infarct border zone, an area crucial for sustaining cardiac function and ensuring long-term recovery [9].

### 2.1. Border Zone Contractility

Contractility can be defined through various methods. Clinically, it is commonly assessed by measuring myocardial shortening between end-diastole and end-systole via echocardiography or cardiac MRI [15]. However, this approach does not fully capture the active force generation capability of myocardial fibers, which directly reflects the heart’s ability to contract and pump blood effectively. In our work, contractility is often specifically defined as the active force or stress produced by myocardial fibers upon electrical stimulation, referred to as T_max_ [9]. This distinction is especially relevant in the infarct border zone, where perfusion might appear normal, yet the actual contractility is severely compromised.

Conventional imaging methods, such as speckle-tracking echocardiography or MRI-based strain analyses, offer valuable regional myocardial deformation data but fall short in directly quantifying the underlying myocardial contractility. To accurately measure contractility, strain data obtained from advanced imaging methods like CSPAMM MRI must be integrated into FE models coupled with optimization algorithms. These algorithms iteratively adjust regional contractility parameters—specifically active fiber stress (T_max_)—within the infarct border zone and remote myocardium until the FE model-predicted strain closely aligns with observed strain measurements. This combined FE modeling approach precisely quantifies the mechanical contributions of infarcted and border zone tissues to overall ventricular dysfunction, providing insights unattainable through traditional imaging alone [11].

### 2.2. Patient-Specific Contractility Mapping

FE modeling thus provides a patient-specific framework to translate imaging-derived strain data into detailed mechanical analyses. These simulations have revealed critical insights, particularly highlighting the significant reduction (approximately 50%) in contractility within the infarct border zone despite normal perfusion—an aspect frequently missed by conventional imaging modalities [9]. Identifying such mechanically impaired regions is essential for optimizing targeted therapeutic interventions, such as myocardial injections or mechanical reinforcements, to halt further deterioration and restore function [16]. FE models, therefore, serve as vital tools for guiding early and personalized intervention strategies aimed at improving outcomes for post-MI patients.

**Figure 2:**
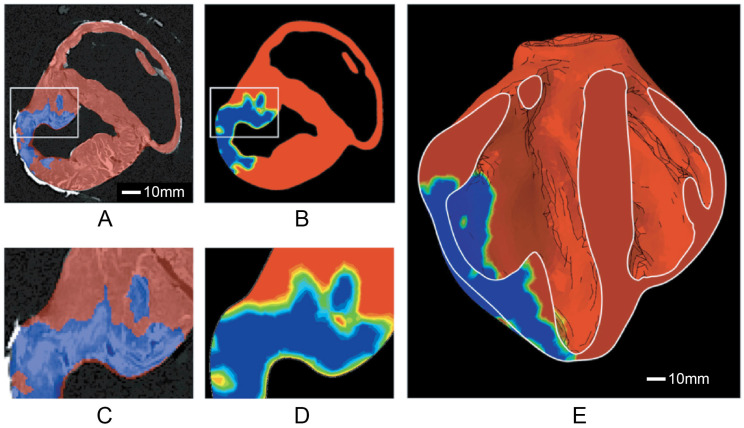
(**A**) Binary segmentation of infarcted (blue) and healthy (red) myocardium on a short-axis MRI from a porcine HF model. (**B**) Corresponding short-axis slice of the FE model showing the interpolated h field, where 0 is blue and 1 is red; intermediate colors (yellow, green) represent the infarct border zone. (**C**) Enlarged view of the region marked in (**A**). (**D**) Enlarged view of the region marked in (**B**). (**E**) Long-axis view of the FE model showing the interpolated h field across the bisected geometry.

**Figure 3:**
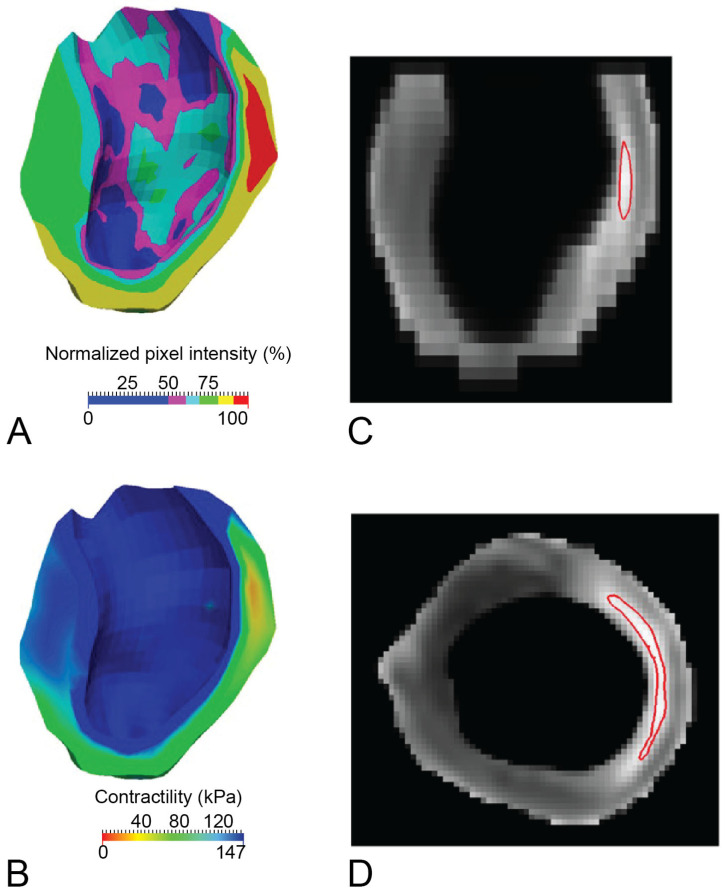
(**A**) Viability map captured during early diastole. Healthy myocardium, indicated by low pixel intensity, appears in blue, while infarcted tissue, with high pixel intensity, appears in red. (**B**) Personalized contractility map derived via numerical optimization. Color mapping is inverted relative to the viability map to maintain consistent regional identification: healthy areas (high contractility) appear in blue, and infarcted areas (low contractility) in red. Since the core infarct region is small, the area with zero contractility (red) is also limited. (**C**) Long-axis contour plot of 95% normalized pixel intensity. Based on material optimization, this delineates regions with less than 10% of the contractility found in remote myocardium. (**D**) Corresponding contour plot in a midventricular short-axis slice, as shown in (**C**).

Figure 2 demonstrates contractility visualization within the infarct border zone in a porcine HF model, highlighting the mechanical transition from infarcted to healthy myocardium. Such mappings are invaluable for pinpointing critical regions that would benefit most from targeted interventions [17]. Similarly, Figure 3 presents a patient-specific contractility mapping approach, illustrating how integrating non-invasive imaging with FE modeling facilitates precise quantification of myocardial stiffness and contractility prior to therapeutic intervention. These modeling tools empower clinicians to tailor personalized treatments such as myocardial injections or surgical reshaping to individual patient needs [15].

Ultimately, these FE models are transforming clinical capabilities in diagnosing myocardial dysfunction, personalizing therapeutic strategies, and significantly enhancing outcomes for MI patients. As FE-based predictive methodologies continue to evolve and integrate artificial intelligence (AI) techniques, the potential for near real-time, patient-specific treatment planning to optimize mechanical recovery and long-term cardiac function becomes increasingly attainable [18].

## 3. Personalized Heart Failure Interventions: Geometry and Mechanical Unloading

Heart failure with reduced ejection fraction (HFrEF) is characterized by progressive dilation of the LV, resulting in increased sphericity [19]. This alteration in geometry severely compromises both myocardial contractility and ventricular filling efficiency, subsequently reducing cardiac output and overall cardiac performance [10]. Effective therapeutic interventions must, therefore, not only target ventricular dilation but also restore optimal ventricular geometry to improve both systolic and diastolic mechanics, thereby enhancing clinical outcomes [10].

### 3.1. Mechanical Circulatory Support Devices (LVADs)

Mechanical circulatory support devices, such as LV assist devices (LVADs), are extensively employed to reduce myocardial wall stress and provide hemodynamic support in advanced HF cases [20]. However, careful device tuning is critical to prevent right ventricular (RV) failure, commonly arising from adverse interventricular septal shifts. Excessive unloading of the LV can lead to abnormal leftward displacement of the septum, compromising RV geometry and increasing afterload. FE modeling serves as an invaluable predictive tool to anticipate and mitigate such complications before device implantation. Unlike traditional experimental methods constrained by ethical and physiological limitations, FE modeling accurately simulates patient-specific hemodynamic responses under various loading conditions, facilitating optimization of LVAD settings to enhance patient outcomes [21].

**Figure 4:**
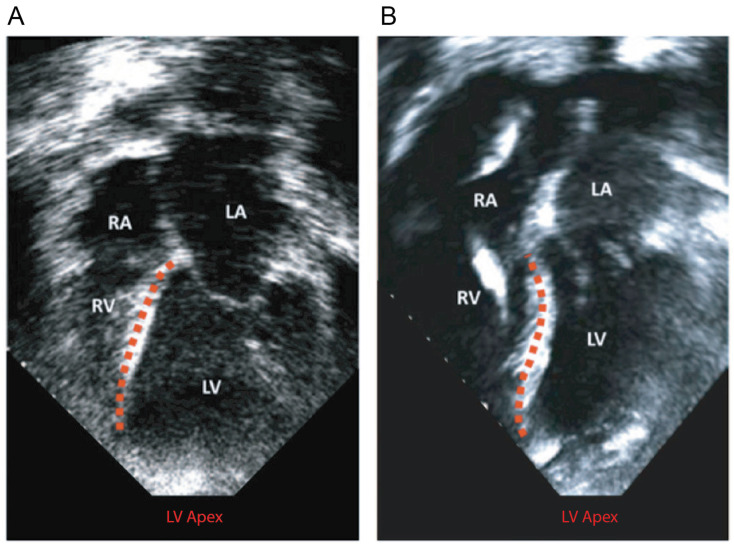
(**A**) A patient with low LVAD rotational speed (RPM) showing a normal, right-shifted interventricular septum (outlined in red), compared to (**B**) a patient with a significantly left-shifted septum (also outlined in red). Abbreviations: LV—left ventricle; RV—right ventricle; LA—left atrium; RA—right atrium.

Figure 4 illustrates the impact of LVAD flow rates on septal positioning, demonstrating a direct correlation between increased flow rates and pronounced leftward septal displacement, thereby heightening the risk of RV failure. These visualizations underscore the necessity of precise LVAD parameter adjustments to balance mechanical unloading benefits against potential adverse outcomes [21].

### 3.2. Historical Surgical Approaches: Limitations of Volume Reduction

The significance of maintaining optimal LV geometry becomes particularly evident when examining historical surgical interventions aimed at reducing ventricular dilation. Procedures such as the Batista ventriculectomy, involving excision of LV wall segments to reduce chamber volume, initially appeared promising but ultimately exhibited high rates of ventricular re-dilation and clinical deterioration within months post-surgery [22,23]. Similarly, surgical ventricular restoration (SVR), designed to reshape the ventricle by excluding dysfunctional segments, often failed to restore favorable geometry, inadvertently increasing ventricular sphericity and further compromising cardiac function [10]. FE modeling has critically elucidated these shortcomings by predicting structural and functional outcomes of these interventions, emphasizing the inadequacy of mere volume reduction without sustained structural support. These findings are summarized in Table 1, which compares traditional volume-reduction surgeries (Batista ventriculectomy and SVR) with novel myocardial reinforcement strategies (hydrogel injection therapy).

**Figure 5:**
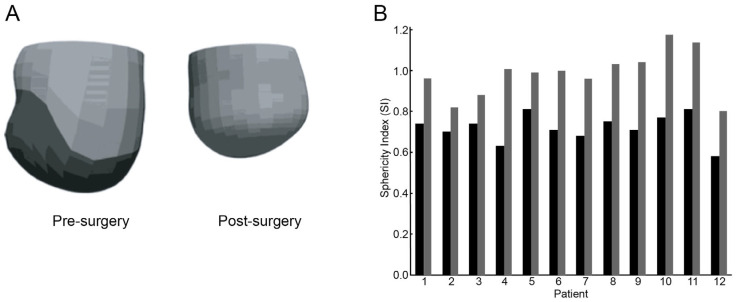
(**A**) Representative FE model of the LV from Patient 1, shown pre-surgery (left) and post-surgery (right). In the preoperative model, dark and light areas represent the infarcted and remote myocardial regions, respectively. (**B**) Sphericity index (SI) measurements for 12 patients are displayed as solid bars before surgery and shaded bars after surgery.

Figure 5 illustrates the predictive power of FE modeling, displaying pre- and post-surgical simulations of LV infarcted and remote myocardial regions. Accompanying sphericity index measurements from 12 patients provide quantitative evidence of how modifications in ventricular geometry correlate with clinical outcomes. These simulations underscore FE modeling’s capacity to assist clinicians in anticipating the biomechanical impacts of proposed surgical alterations, thereby preventing unintended exacerbation of LV dysfunction [10].

### 3.3. Novel Reinforcement Strategies: Hydrogel Injection Therapy

Recognizing the limitations of traditional volume-reduction techniques has motivated the exploration of novel approaches that reinforce rather than remove myocardial tissue. One promising innovation involves injecting alginate-based hydrogel polymers directly into the LV myocardium, offering structural reinforcement. A notable implementation of this strategy, Algisyl injection therapy, mitigates progressive ventricular dilation while maintaining myocardial contractility. This approach effectively prevents additional geometric deterioration without negatively impacting mechanical performance [24]. FE modeling has played a crucial role in validating the benefits of hydrogel injections, demonstrating improved ventricular geometry and enhanced systolic function. These findings emphasize the essential contribution of computational modeling in developing patient-specific interventions, ensuring therapeutic strategies not only correct anatomical abnormalities but also optimize cardiac biomechanics for improved clinical outcomes [25].

**Table 1:** Comparison of Traditional Volume-Reduction Surgeries and Novel Myocardial Reinforcement Strategies.

Strategy	Mechanism	FE Modeling Insights	Clinical Outcomes
Batista Ventriculectomy	Surgical excision of LV wall segments to reduce chamber volume [23]	Predicts transient reduction in volume and wall stress, with little improvement in LV function due to abnormal stress distribution [26]	Temporary improvements in EF; high recurrence of dilation; poor long-term survival [27]
Surgical Ventricular Restoration (SVR)	Excludes dysfunctional LV segments [10]	FE models show increased sphericity, worsening mechanical efficiency [10]	Modest EF improvement; inconsistent outcomes; high reoperation rates [28]
Alginate-Based Hydrogel Injection	Intramyocardial injection of hydrogel to create midwall structural reinforcement [24]	Predicts improved stress distribution, reduced ESV, and stabilized geometry [25]	Stabilized LV dimensions; improved systolic function; reduced progression of HF [24]

## 4. Hydrogel Injections as a Midwall Passive Constraint for the LV Free Wall

Historically, surgical approaches to managing HFrEF have typically focused on removing or excluding areas of dysfunctional myocardial tissue. However, as evidenced by the limited effectiveness of techniques such as the Batista ventriculectomy [26] and SVR [10], simple volume reduction does not consistently lead to improved cardiac function. Consequently, there has been a growing shift towards therapeutic strategies aimed at reinforcing weakened myocardial tissue, preserving optimal ventricular geometry, and limiting adverse remodeling.

### 4.1. Mechanisms and Clinical Advantages

Passive constraint methods, including biventricular wraps, have been introduced to counteract LV dilation by externally supporting the epicardium. Despite their efficacy in restricting chamber expansion, these devices often impose significant limitations. Encasing both ventricles externally can constrain ventricular motion broadly, potentially compromising both left and right ventricular filling and contraction. Particularly problematic is the restriction of right ventricular (RV) motion, which may inadvertently worsen clinical symptoms in heart failure patients [29].

In contrast, an innovative and increasingly explored alternative involves the intramyocardial injection of alginate-based hydrogels, such as Algisyl therapy, functioning as midwall passive constraints. Unlike external constraint devices, hydrogel injections integrate directly within the myocardial tissue, creating internal scaffolds that provide structural support without impeding RV motion. Due to their inherent flexibility and softness, these hydrogels mimic the mechanical characteristics of non-contractile myocardium, effectively stabilizing LV geometry and preventing progressive dilation while maintaining myocardial contractility [19,25,30]. A critical clinical advantage of this strategy includes its ability to significantly decrease end-systolic volume (ESV), an important prognostic indicator strongly associated with patient outcomes in HF [24]. Experimental and clinical data from animal models show that untreated HFrEF subjects typically experience continuous LV dilation, decreased systolic function, and elevated mortality risk. Conversely, Algisyl-treated subjects demonstrate stabilized ventricular dimensions and improved cardiac performance, highlighting the potential of hydrogel injections as an effective, minimally invasive alternative to traditional surgical methods [25].

### 4.2. Role of FE Modeling

**Figure 6:**
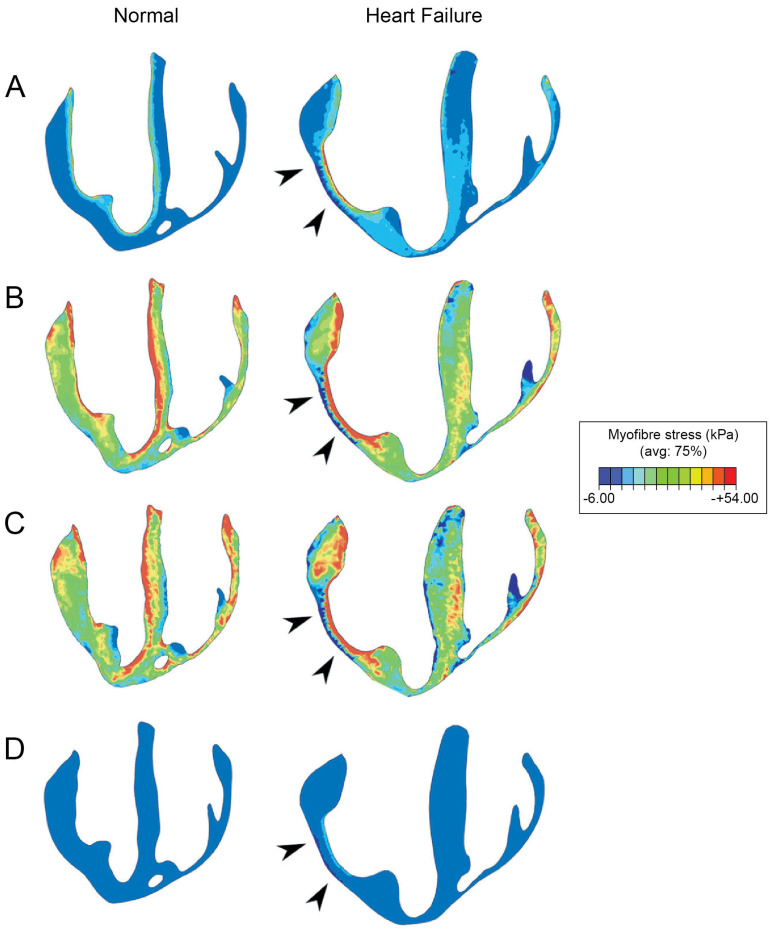
Myofiber stress distributions at four key phases of the cardiac cycle for a representative normal (left column) and HF (right column) porcine heart: (**A**) end-diastole, (**B**) start of ejection, (**C**) end-systole, and (**D**) end-relaxation. Long-axis cross-sections display ventricular wall stress, color-mapped from –6.00 kPa (blue) to +54.00 kPa (red). The infarcted or fibrotic region in the HF heart is indicated by black arrowheads. Asymmetric contour limits were used to allow direct comparison of stress scales across all cardiac phases.

FE modeling plays a pivotal role in optimizing hydrogel injection strategies. These computational simulations facilitate the identification of ideal injection sites, maximizing therapeutic benefits and minimizing mechanical inefficiencies. FE modeling accurately predicts how hydrogel-induced structural modifications influence LV mechanics, ensuring targeted interventions that reinforce myocardial integrity without introducing adverse mechanical effects [25]. Figure 6 illustrates the variations in myocardial wall stress distributions between healthy and failing hearts, demonstrating how altered stress patterns drive pathological remodeling, thinning, and dilation in failing ventricles [17]. By pinpointing regions of increased mechanical stress, FE models effectively guide hydrogel placements, mitigating these deleterious effects.

The distribution and management of myocardial wall stress are central determinants of cardiac remodeling, helping explain the differential outcomes of various HF treatments [31]. Traditional volume reduction procedures, such as those analyzed in the STICH trial, often failed to adequately address the redistribution of myocardial stress, leading to suboptimal clinical outcomes despite reductions in ventricular volume [10,28]. Conversely, interventions like hydrogel injections and optimized LVAD settings, facilitated by FE modeling, restore favorable stress profiles, thereby preventing further adverse remodeling and preserving cardiac function [21,25]. Additionally, the beneficial stress redistribution provided by hydrogel injections has implications for mitigating secondary mitral regurgitation by stabilizing ventricular geometry and preventing pathological displacement of papillary muscles and mitral annular dilation [32].

**Figure 7:**
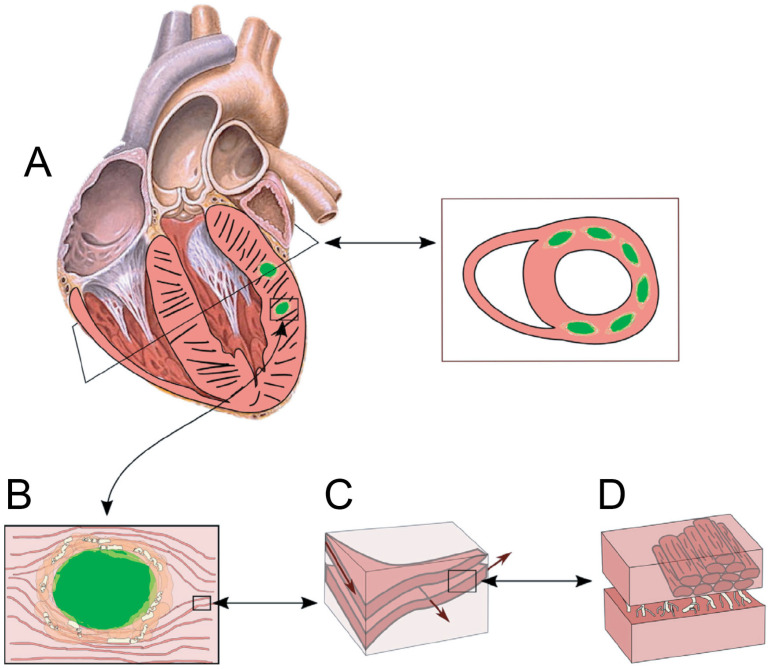
Schematic illustrating the solidified hydrogel and surrounding fibrotic capsule across macro- to microscale views (**A**–**D**). The capsule functions to “glue” adjacent myocytes, forming a circumferential constraint via multiple non-degradable circumferential implants. Panels (**A**,**B**) depict the hydrogel implant and capsule securely linking neighboring myocardial sheets, thereby increasing ventricular stiffness in the circumferential direction. The injection pattern includes 12–14 injections (0.3 mL each), arranged in two rows—one above and one below the mid-ventricular plane—extending from the anterior to the posterior wall between the base and apex. Panels (**C**,**D**) illustrate the orthotropic ultrastructure of the myocardium.

Figure 7 provides a schematic representation of hydrogel injections, illustrating how the injected material integrates into myocardial tissue to form an internal midwall reinforcement. Unlike external wraps, this targeted reinforcement approach preserves myocardial contractility and ventricular motion, effectively halting adverse dilation [25]. Moreover, FE modeling studies have demonstrated that hydrogel injections do not increase arrhythmic risks, further emphasizing their clinical utility in HF management [25].

### 4.3. Clinical Translation and Challenges

Despite the clear promise of hydrogel-based myocardial reinforcement, translating these strategies into routine clinical practice has faced several hurdles. Early clinical trials often suffered from suboptimal patient selection, with therapies applied too late in disease progression for patients to fully benefit [25]. Furthermore, technical challenges associated with minimally invasive hydrogel delivery, particularly to complex anatomical regions such as the interventricular septum, have significantly impeded broader clinical adoption [25]. These delivery challenges occasionally resulted in severe adverse events, further limiting therapeutic acceptance [24]. Moving forward, continued advancement in FE modeling will be crucial for refining patient selection criteria and developing targeted, minimally invasive delivery techniques, thereby optimizing clinical outcomes and maximizing the therapeutic potential of hydrogel-based myocardial reinforcement strategies.

## 5. AI-Enhanced FE Models for Real-Time Clinical Applications

Traditional FE modeling is limited by significant computational demands, often taking hours or even days to produce meaningful results, thus limiting its utility for immediate clinical decision-making [13]. In clinical settings where rapid decision-making is essential, these delays hinder the timely implementation of personalized therapeutic interventions.

### 5.1. AI-Driven Surrogate Models

**Figure 8:**
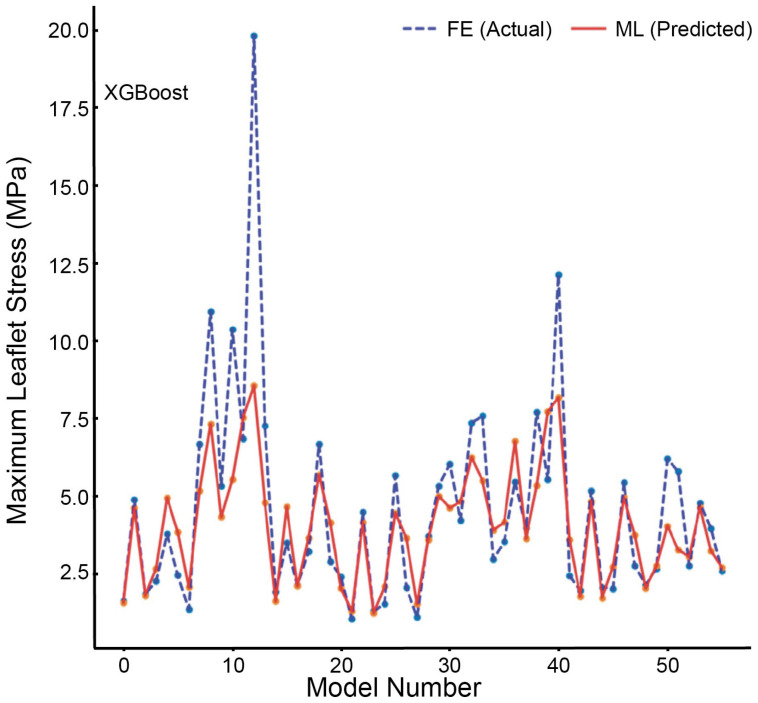
Maximum leaflet von Mises stress values for the test set, as obtained from FE simulations (ground truth) and XGBoost predictions. The machine learning model (XGBoost) shows strong agreement with the FE-derived values, despite requiring significantly less computation time—1 s for the ML model compared to 6 h for conventional FE analysis.

To address this issue, artificial intelligence (AI)-based surrogate models, trained on extensive datasets of precomputed FE simulations, have emerged as a transformative solution, offering rapid, near-instantaneous predictions of therapeutic outcomes [33]. Machine learning (ML) algorithms, such as XGBoost, have demonstrated the capability to accurately approximate complex cardiac stress distributions and mechanical behaviors by training on large sets of pre-simulated FE data [13]. Figure 8 highlights the potential of such AI-driven surrogate models in expediting FE simulation processes for clinical applications. The graph compares the maximum leaflet von Mises stress values derived from traditional FE simulations with predictions obtained using an XGBoost machine learning model. Remarkably, the ML approach delivers stress predictions closely matching traditional FE-derived values while drastically reducing computation time—from approximately six hours down to a mere second. This efficiency underscores the significant potential of AI-enhanced modeling to streamline stress analysis for real-time clinical decision-making in structural heart disease [13].

### 5.2. Clinical Integration and Rapid Decision-Making

Advancing towards practical real-time clinical implementation, a promising direction involves the creation of comprehensive databases comprising precomputed FE simulations across a broad spectrum of therapeutic scenarios [13]. Clinicians can rapidly compare patient-specific anatomical and pathological characteristics against the database, swiftly identifying the most relevant precomputed simulations. This method facilitates rapid therapy selection without necessitating time-intensive, real-time computations [33]. Integrating AI-driven predictions with these extensive precomputed FE datasets enables clinicians to promptly evaluate the mechanical implications of diverse interventions, essential for precise biomechanical optimization required in procedures such as LVAD parameter tuning [21], targeted hydrogel injections [25], and SVR techniques [10]. By moving from computationally intensive real-time FE analyses to AI-enhanced predictive frameworks, personalized computational modeling becomes practically achievable within clinical HF management.

### 5.3. Balancing Accuracy and Efficiency

Notably, FE models do not require exhaustive perfection to yield clinically valuable insights [17]. Traditional FE approaches typically aim to represent all six components of myocardial strain; however, for clinical purposes, accurately predicting circumferential and longitudinal strain often suffices for evaluating novel surgical and interventional therapies [17]. This pragmatic approach allows AI-enhanced FE models to prioritize computational efficiency and clinical applicability without compromising significant accuracy [13]. As illustrated by Figure 8, AI-driven predictions of critical stress distributions closely align with comprehensive FE calculations, thereby supporting real-time clinical decision-making.

Integrating AI-powered FE models into clinical practice significantly enhances therapy personalization and adaptive intervention strategies. Rapidly simulating outcomes of specific treatments, such as LVAD settings adjustments [21], precise hydrogel injection placements [25], or tailored surgical planning [10], greatly enhances clinical decision-making capabilities, ultimately improving patient outcomes. Bridging the gap between computational modeling and practical clinical utilization, AI-enhanced FE modeling has the potential to profoundly transform the clinical management of structural heart disease.

## 6. Conclusions and Key Insights for Clinical Translation

FE modeling has transitioned from a research-centric approach to an increasingly vital tool in clinical practice, significantly enhancing the precision and effectiveness of surgical and interventional strategies. Crucially, these models need not achieve perfect accuracy to provide clinically valuable insights [17]. Even streamlined, patient-specific simulations can meaningfully inform and refine therapeutic strategies prior to clinical intervention, thereby improving precision and minimizing procedural risks.

One of the most impactful contributions of FE modeling in clinical care is its capability to optimize therapy selection and placement. For instance, FE simulations facilitate the identification of optimal sites for hydrogel injections, ensuring structural reinforcement while minimizing arrhythmic risks [25]. Likewise, FE modeling plays a critical role in fine-tuning LVAD settings [21] and refining surgical ventricular reconstruction techniques [10], ensuring mechanical efficiency and preventing unintended adverse cardiac remodeling.

Looking ahead, the integration of artificial intelligence (AI) with FE modeling represents a transformative advancement, enabling real-time, patient-specific therapeutic decision-making. AI-powered surrogate models will allow clinicians to rapidly simulate various intervention outcomes, embedding FE modeling deeply within routine clinical workflows and transforming it from a research tool into an indispensable clinical resource [13]. As these methodologies continue to evolve, they hold immense potential to revolutionize structural heart disease treatment by offering truly personalized, biomechanically informed therapy planning.
