# Correction: Hoang, P.; Guccione, J. Finite Element Modeling in Left Ventricular Cardiac Biomechanics: From Computational Tool to Clinical Practice. *Bioengineering* 2025, *12*, 913

**DOI:** 10.3390/bioengineering13010006

**Published:** 2025-12-22

**Authors:** Patrick Hoang, Julius Guccione

**Affiliations:** 1 School of Medicine, University of California San Francisco, San Francisco, CA 94143, USA; patrick.hoang@ucsf.edu; 2 Medical Scientist Training Program, University of California San Francisco, San Francisco, CA 94143, USA; 3 Department of Surgery, University of California San Francisco, San Francisco, CA 94143, USA

## Improper Citation of a Reused Figure

In the original publication [1], a reused figure was cited incorrectly (Figure 1). Although the authors included the source within the manuscript, they did not provide an explicit attribution line in the figure caption. To address this issue, the caption of Figure 1 needs to be updated to include the required attribution. The corrected caption for Figure 1 is provided below.

**Figure 1**. Human heart models have increased in complexity over time. Simplified geometries—such as idealized cylindrical, elliptical LV, and elliptical biventricular models—enable analytical closed-form expressions for fiber orientation. In contrast, more anatomically realistic models, including axisymmetric, LV, biventricular, and whole-heart geometries, are derived from actual human heart anatomy and fiber architecture. Reproduced from [12], under the Creative Commons CC BY 4.0 license.

## Missing Funding Information

In the original publication [1], the authors did not enclose the funding information. The correct funding statement is shown below:

**Funding:** This study was funded by NIH grant 4R44HL169034-02.

The authors state that the scientific conclusions are unaffected. This correction was approved by the Academic Editor. The original publication has also been updated.
